# Congestion of the Brain

**Published:** 1842-07-16

**Authors:** 


*Congestion of the Brain.*—A. W., a coloured man, æt. 32, entered the medical ward May 31st. He was taken ill four weeks previous with "sick headache," and had a blister applied to the nucha. The above was all the history that could be obtained from him. When he entered the ward his state was as follows: Expression stupid and somnolent; was aroused with difficulty; answered questions very tardily, and sometimes incoherently; memory was almost destroyed; eyes were suffused; conjunctiva injected; slight subsultus; tongue dry and white; respiration natural, 24; pulse 96; skin moist and cool. He had no pain; no abdominal tenderness, and no tympanitis. His urine was straw coloured, clear, and exhibited no deposit by the addition of nitric acid. He was treated with occasional cupping to the nucha, and half an ounce of the oleum terebinthinæ in four or five ounces of mucilage was injected up the rectum night and morning. He also was directed a warm pediluvium frequently. He continued in about the same condition for two days, at which time he commenced improving, and was pronounced convalescent on the 6th of June, from which time this report dates.

